# Rewiring of embryonic glucose metabolism via suppression of PFK-1 and aldolase during mouse chorioallantoic branching

**DOI:** 10.1242/dev.138545

**Published:** 2017-01-01

**Authors:** Hidenobu Miyazawa, Yoshifumi Yamaguchi, Yuki Sugiura, Kurara Honda, Koki Kondo, Fumio Matsuda, Takehiro Yamamoto, Makoto Suematsu, Masayuki Miura

**Affiliations:** 1 Department of Genetics, Graduate School of Pharmaceutical Sciences, The University of Tokyo, Bunkyo-ku, Tokyo 113-0033, Japan; 2 Precursory Research for Embryonic Science and Technology, Japan Science and Technology Agency, Chiyoda-ku, Tokyo 102-0075, Japan; 3 Department of Biochemistry, Keio University School of Medicine, Shinjuku-ku, Tokyo 160-8582, Japan; 4 Department of Bioinformatic Engineering, Graduate School of Information Science and Technology, Osaka University, Suita, Osaka 565-0871, Japan; 5 Agency for Medical Research and Development-Core Research for Evolutional Medical Science and Technology (AMED-CREST), Japan Agency for Medical Research and Development, Chiyoda-ku, Tokyo 100-1004, Japan

**Keywords:** Energy metabolism, Chorioallantoic branching, Lin28a, Phosphofructokinase-1, Mouse, Imaging mass spectrometry

## Abstract

Adapting the energy metabolism state to changing bioenergetic demands is essential for mammalian development accompanying massive cell proliferation and cell differentiation. However, it remains unclear how developing embryos meet the changing bioenergetic demands during the chorioallantoic branching (CB) stage, when the maternal-fetal exchange of gases and nutrients is promoted. In this study, using metabolome analysis with mass-labeled glucose, we found that developing embryos redirected glucose carbon flow into the pentose phosphate pathway via suppression of the key glycolytic enzymes PFK-1 and aldolase during CB. Concomitantly, embryos exhibited an increase in lactate pool size and in the fractional contribution of glycolysis to lactate biosynthesis. Imaging mass spectrometry visualized lactate-rich tissues, such as the dorsal or posterior neural tube, somites and head mesenchyme. Furthermore, we found that the heterochronic gene *Lin28a* could act as a regulator of the metabolic changes observed during CB. Perturbation of glucose metabolism rewiring by suppressing *Lin28a* downregulation resulted in perinatal lethality. Thus, our work demonstrates that developing embryos rewire glucose metabolism following CB for normal development.

## Abstract (precis)

**Highlighted article:** Metabolic remodelling during E8.5 to E10.5 in mouse redirects glucose carbon into the pentose phosphate pathway, a process partially regulated by Lin28a.

## INTRODUCTION

Understanding how cellular metabolism is coordinated with various biological processes at the tissue or organismal level has been a challenge for modern biology (Boroughs and DeBerardinis, 2015; Pavlova and Thompson, 2016; Vander Heiden et al., 2009). Recent metabolic research in the cancer and stem cell fields has highlighted the importance of cellular metabolism in dictating cell proliferation and cell differentiation; actively dividing cells favor glycolysis for efficient biomass production, whereas terminally differentiated cells mainly rely on oxidative phosphorylation (OXPHOS) for efficient energy production (Agathocleous and Harris, 2013; Christofk et al., 2008; Folmes et al., 2011; Vander Heiden et al., 2009).

This also seems to be the case during development, when cell proliferation and cell differentiation occur concurrently (Agathocleous et al., 2012; Tennessen et al., 2011). In flies, for example, aerobic glycolysis is activated during larval development for efficient biomass production, thereby achieving a dramatic increase in body mass (Tennessen et al., 2011). In mammals, it is proposed that the center of embryonic energy metabolism shifts from glycolysis to OXPHOS during the transition from gastrulation to the neurulation stage [embryonic day (E)6-9 in mice] (Clough, 1985; Clough and Whittingham, 1983). These stages following gastrulation are accompanied by alterations in the intrauterine environment of embryos, namely, the establishment of vitelline circulation and, subsequently, chorioallantoic branching (CB) (Arora and Papaioannou, 2012; McGrath et al., 2003; Rossant and Cross, 2001; Zohn and Sarkar, 2010). CB facilitates the uptake/circulation of gases and nutrients between the mother and the fetus and impacts the growth and development of the whole embryo (Luo et al., 1997). These changes require precise coordination between embryonic energy metabolism and the maternal environment. Embryos harboring mutations in key regulators of energy metabolic pathways exhibit defects around these stages of development (Bamforth et al., 2001; Davis et al., 1993; Iyer et al., 1998; Larsson et al., 1998).

Although studies in the late 20th century proposed that glycolysis decreases and OXPHOS increases following mammalian CB, this widely accepted notion should be reconsidered owing to the technical limitations that existed during that era for studying cellular energy metabolism within developing embryos. Those studies measured only lactate and CO_2_, which were secreted from cultured embryos (Clough and Whittingham, 1983; Tanimura and Shepard, 1970), and therefore might not capture the complete picture of metabolic change *in vivo*. Furthermore, a simple shift from glycolysis to OXPHOS does not explain how mammalian embryos adapt their energy metabolism state to increasing metabolic demands for biomass production and for energy production in order to support cell proliferation, cell differentiation, and morphogenesis during the neurulation stage.

In this study, we conducted a global metabolome analysis of intracellular metabolites by taking advantage of recent technical advances in mass spectrometry, including imaging mass spectrometry. These state of the art techniques revealed a novel perspective on the rewiring of embryonic glucose metabolism during mouse CB. Furthermore, we demonstrated that downregulation of the heterochronic gene *Lin28a* could be a component of the developmental programs mediating metabolic rewiring during this stage of development.

## RESULTS

### Transition of embryonic metabolome profiles during CB

To reveal how the embryonic metabolome profiles change during CB (E8.5 to E10.5 in mice) (Rossant and Cross, 2001), we performed a metabolome analysis of embryos at E8.5 [somite stage (ss) 10-12], E9.5 (ss 24-26) and E10.5 (ss 35-37) using capillary electrophoresis-mass spectrometry (CE-MS) (Soga et al., 2006). This allowed us to construct the first comprehensive metabolome of CB stage embryos.

We detected 219 intracellular metabolites, and discovered that the embryonic metabolome profiles clearly changed from E8.5 to E10.5 (Fig. S1A, Table S1). A supervised statistical analysis by partial least-square discriminant analysis (PLS-DA) revealed a statistically significant difference between the developmental stages (Fig. S1A). Whereas PLS2 discriminated the difference between E8.5/E10.5 and E9.5 embryos, PLS1 clearly discriminated the difference between E8.5, E9.5 and E10.5 embryos. Metabolites involved in glycolysis and the tricarboxylic acid (TCA) cycle ranked highly among the metabolites that contributed the most to the separation within the PLS1 analysis and whose amounts changed significantly between these stages (Fig. S1B).

### Increased pool size of lactate and TCA cycle metabolites following CB

**Fig. 1.**
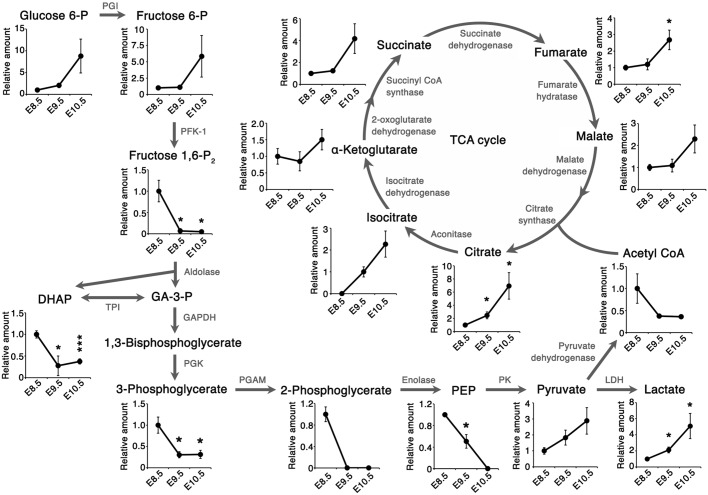
**Metabolome analysis during chorioallantoic branching.** Intracellular metabolite levels were quantified by CE-MS analysis and normalized to the total amount of protein extracted from the embryos. Relative amount refers to the amount of intracellular metabolites in the TCA cycle and glycolysis relative to E8.5 embryos. Data are represented as mean±s.d. *n*=3 independent sample replicates. *P<0.05, ***P<0.001, versus E8.5 embryos (two-tailed Welch's *t*-test). E8.5, somite stage (ss) 10-12; E9.5, ss 24-26; E10.5, ss 35-37. DHAP, dihydroxyacetone phosphate; GA-3-P, glyceraldehyde 3-phosphate; PEP, phosphoenol pyruvate; PGI, phosphoglucose isomerase; PFK-1, phosphofructokinase-1; TPI, triose phosphate isomerase; GAPDH, glyceraldehyde 3-phosphate dehydrogenase; PGK, phosphoglycerate kinase; PGAM, phosphoglycerate mutase; PK, pyruvate kinase; LDH, lactate dehydrogenase.

**Fig. 2.**
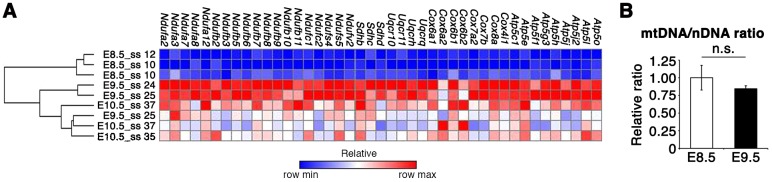
**Expression of mitochondrial ETC genes increases from E8.5 to E9.5.** (A) Heatmap representation of mitochondrial electron transport chain (ETC)-related genes that were differentially expressed between E8.5 and E9.5 in a microarray analysis. *n*=3 independent sample replicates. Fold change ≥1.5 versus E8.5. *P*<0.05 versus E8.5 (one-way ANOVA with Tukey's post-hoc test). Multiple testing correction, Benjamini–Hochberg FDR; hierarchical clustering with Manhattan distance. E8.5, ss 10-12; E9.5, ss 24-25; E10.5, ss 35-37. Components of each complex were picked up by referring to gene ontology terms. (B) Quantification of the mitochondrial DNA (mtDNA)/nuclear DNA (nDNA) ratio by qPCR. No statistically significant difference (n.s.; two-tailed Welch's *t*-test) was observed between E8.5 (ss 10-12) and E9.5 (ss 23-24); *n*=7 and *n*=4 independent sample replicates. Data are represented as mean±s.d.

Although glucose metabolism is known to be crucial for embryonic development during CB (Hunter and Tugman, 1995; Yang et al., 2013), it remains to be fully understood how embryonic glucose metabolism changes at this stage. Our metabolome data showed that intracellular metabolites in the TCA cycle increased significantly from E8.5 to E10.5 (Fig. 1). The increase in TCA cycle metabolite abundance was accompanied by increased expression of mitochondrial electron transport chain (ETC) genes from E8.5 to E9.5 (Fig. 2A). This increased gene expression was not due to the increased number of mitochondria (Fig. 2B). Consistent with the results of previous studies, these results imply that developing embryos prime the ETC for efficient ATP production through OXPHOS during the CB stage (Mackler et al., 1971; Shepard et al., 1998). Interestingly, although previous studies suggested that glycolysis is decreased at this stage (Clough and Whittingham, 1983), the amount of intracellular lactate, which is the end product of glycolysis, also increased significantly from E8.5 to E10.5 (Fig. 1).

### Tissue distribution of glycolytic and TCA cycle intermediates within embryos revealed by imaging mass spectrometry

**Fig. 3.**
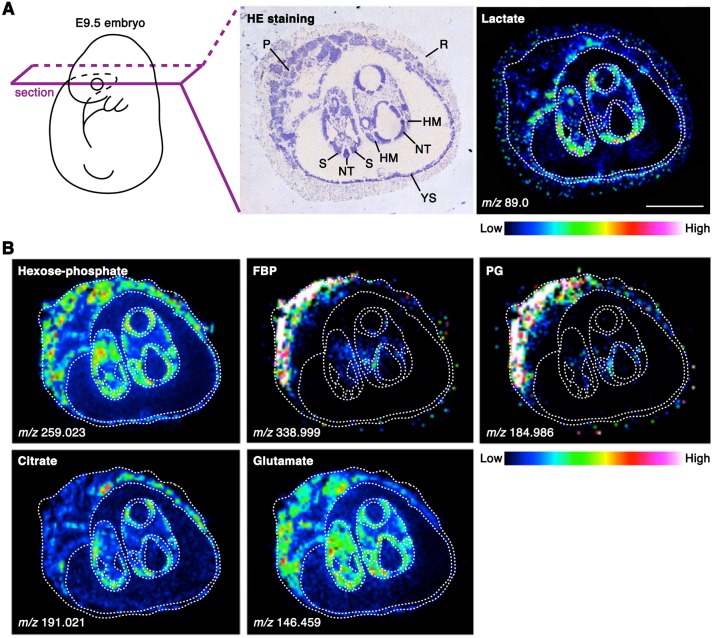
**Tissue distribution of glycolytic and TCA cycle intermediates revealed by MALDI-IMS.** (A) MALDI-IMS analysis of E9.5 embryos. A higher level of lactate (C_3_H_6_O_3_) was observed in the dorsal or posterior neural tube, somites and head mesenchyme than in other tissues, such as extraembryonic tissues. *n*=3 independent sample replicates. (B) MALDI-FT-ICR-MS imaging of E9.5 embryos. The distributions of other glycolytic intermediates including hexose phosphate (C_6_H_13_O_9_P), fructose bisphosphate (FBP; C_6_H_14_O_12_P_2_) and phosphoglycerate (PG; C_3_H_7_O_7_P) are visualized. The localizations of citrate (C_6_H_8_O_7_) and glutamate (C_5_H_9_NO_4_) are also shown. *n*=2 independent sample replicates. HM, head mesenchyme; NT, neural tube; P, placenta; R, Reichert's membrane; S, somite; YS, yolk sac. Scale bar: 1 mm.

We next explored which embryonic structures contribute to the increased lactate level during CB. We investigated the distribution of lactate within E9.5 embryos using matrix-assisted laser desorption/ionization-imaging mass spectrometry (MALDI-IMS), a useful method for visualizing the spatial distribution of individual biomolecules at a spatial resolution of ∼30 µm pitch on intact tissues (Bailey et al., 2015; Hamilton et al., 2015; Sugiura et al., 2014). Fresh frozen embryos were sectioned and analyzed by MALDI-IMS. Whereas hexose phosphate (glucose 1-phosphate, glucose 6-phosphate and fructose 6-phosphate) and glutamate (an abundant amino acid in the uterus) tended to be distributed evenly, higher levels of lactate and citrate were observed within specific tissues, including the dorsal or posterior neural tube, somites and head mesenchyme, as compared with such as the extraembryonic tissues (Fig. 3). These observations suggest that cells within multiple embryonic tissues actively produce lactate and citrate at E9.5.

### Increased contribution of glycolysis to lactate production during CB

**Fig. 4.**
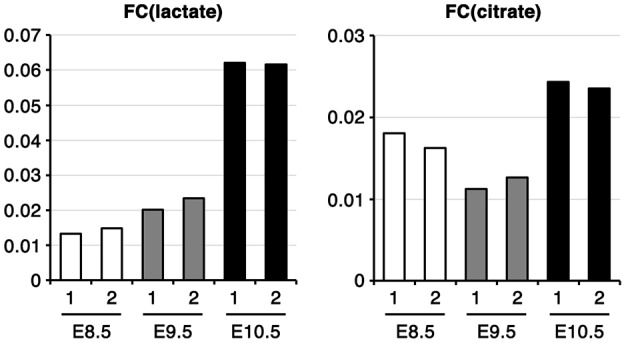
**The fractional contribution of glycolysis to lactate biosynthesis increases during CB.** Fractional contribution of ^13^C from ^13^C_6_-glucose to lactate [FC(lactate)] or citrate [FC(citrate)] *in vivo*. Embryos were collected 60 min after ^13^C_6_-glucose administration. 1 and 2 are independent samples. E8.5, ss 8-12; E9.5, ss 22-26; E10.5, ss 32-36.

Although the results of the above analyses represent snapshots of metabolome profiles, the observed changes in metabolite abundance do not necessarily reflect the flow of each metabolic pathway. To understand changes in the carbon flow of glycolysis and the TCA cycle during development, we traced and compared the metabolic fate of glucose using a fully labeled form (^13^C_6_-glucose) in CB stage embryos *in vivo*. ^13^C_6_-glucose was administered to pregnant mice intravenously, and the amounts of ^13^C-labeled and non-labeled lactate/citrate within embryos were quantified. Changes in the metabolic fate of glucose can be described by the fractional contribution (FC) of ^13^C from ^13^C_6_-glucose to citrate [FC(citrate)] or to lactate [FC(lactate)] (Buescher et al., 2015). FC(citrate) and FC(lactate) were calculated based on a mass distribution vector (MDV) describing the enrichment of each isotopomer (Fig. 4, Fig. S2). Whereas FC(citrate) did not dramatically change, FC(lactate) tended to increase dramatically from E8.5 to E10.5. These results indicate that, during CB, there is an increase in the proportion of glucose-derived lactate in the total lactate pool, implying increased glycolytic lactate production.

### Rewiring of embryonic glucose metabolism via suppression of PFK-1/aldolase

Another remarkable change revealed by our metabolome analysis was that the amount of intracellular metabolites downstream of the reaction catalyzed by phosphofructokinase-1 (PFK-1) decreased dramatically from E8.5 to E9.5 (Fig. 1). In E9.5 embryos, the decreased glycolytic intermediates (fructose bisphosphate and phosphoglycerate) tended to be less detectable within the embryo proper as compared with within the placenta (Fig. 3). Such decreases can be caused by the decreased production or increased consumption of glycolytic intermediates downstream of the PFK-1 reaction.

**Fig. 5.**
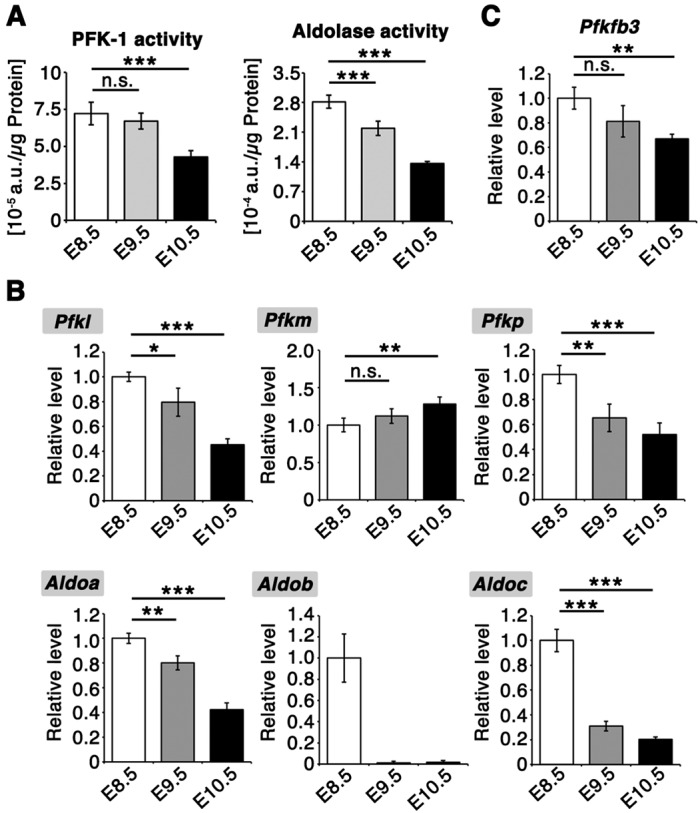
**Suppression of PFK-1/aldolase accompanies glucose metabolism rewiring.** (A) Enzymatic activity assays of PFK-1 and aldolase in whole-embryo lysates. Activities were normalized to the total protein amount in the lysates. *n*=7 independent sample replicates. ***P<0.001 versus E8.5; n.s., not significant (two-tailed Welch's *t*-test). E8.5, ss 8-10; E9.5, ss 25-26; E10.5, ss 35-36. The activities of both PFK-1 and aldolase decreased significantly from E8.5 to E10.5. (B,C) qPCR analysis of glycolytic enzymes. *Actb* was used as an internal control. *Aldob* expression was below the detection limit in three out of four samples at E9.5 and E10.5. *n*=4 independent sample replicates. *P<0.05, **P<0.01, ***P<0.001 versus E8.5; n.s., not significant (two-tailed Welch's *t*-test). E8.5, ss 10; E9.5, ss 23-26; E10.5, ss 34-36. Data are represented as mean±s.d.

We then examined the enzymatic activities of both PFK-1 and aldolase in embryos and found that they were significantly reduced from E8.5 to E10.5 (Fig. 5A). The reduction in the enzymatic activities of PFK-1 and aldolase was partially due to decreased expression of the genes that encode these enzymes [*Pfkl*, *Pfkp*, aldolase A (*Aldoa*), aldolase B (*Aldob*) and aldolase C (*Aldoc*)] (Fig. 5B). In addition, we observed a significant decrease in *Pfkfb3*, which encodes an enzyme that preferentially produces fructose 2,6-bisphosphate (F-2,6-BP), the most potent activator of PFK-1 (Mor et al., 2011), from E8.5 to E10.5 (Fig. 5C). These results suggest that the reactions catalyzed by PFK-1 and aldolase in the glycolytic pathway are suppressed during CB.

Aldolase activity was suppressed to a greater degree than that of PFK-1 (Fig. 5A), implying that regulation of aldolase might play a key role in the regulation of glucose metabolism rewiring during this period. Whole-mount *in situ* hybridization analysis revealed that *Aldoa*, which encodes the major aldolase present during CB, was ubiquitously expressed at E8.5. Notably, it became localized to the somites and the posterior part of the embryo as development proceeded (Fig. S3), implying that the rewiring of glycolysis occurs along the rostral-to-caudal axis of the embryo.

### Increased pool size of the pentose phosphate pathway (PPP) and the biosynthetic pathway during CB

**Fig. 6.**
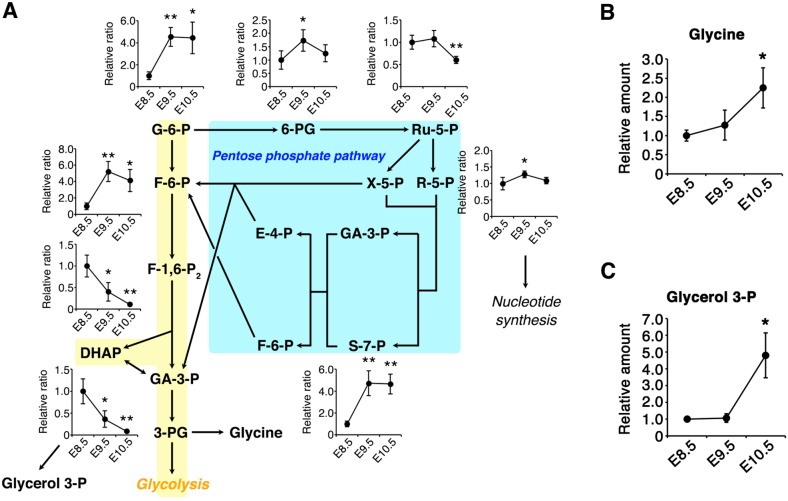
**The increased pool size of the pentose phosphate pathway (PPP) during CB.** (A) PPP metabolites were quantified by IC-MS. Area values were normalized to the amount of protein extracted from the embryos, and the relative ratios to E8.5 embryos are shown. *n*=4 independent sample replicates. *P<0.05, **P<0.01 versus E8.5 (two-tailed Welch's *t*-test). E8.5, ss 8-11; E9.5, ss 24-25; E10.5, ss 34-35. G-6-P, glucose 6-phosphate; F-6-P, fructose 6-phosphate; F-1,6-P_2_, fructose 1,6-bisphosphate; DHAP, dihydroxyacetone phosphate; GA-3-P, glyceraldehyde 3-phosphate; 6-PG, 6-phosphogluconate; Ru-5-P, ribulose 5-phosphate; R-5-P, ribose 5-phosphate; X-5-P, xylulose 5-phosphate; S-7-P, sedoheptulose 7-phosphate; E-4-P, erythrose 4-phosphate. (B,C) Amounts of glycerol 3-phosphate and glycine determined by CE-MS were normalized to the total amount of protein extracted from the embryos, and the relative ratios to E8.5 embryos are shown. *n*=3 independent sample replicates. *P<0.05 versus E8.5 embryos (two-tailed Welch's *t*-test). E8.5, ss 10-12; E9.5, ss 24-26; E10.5, ss 35-37. Data are represented as mean±s.d.

We next aimed to assess how suppression of PFK-1/aldolase affects the pool sizes of other metabolic pathways branching from the glycolytic pathway. Suppression of PFK-1/aldolase reactions can result in glucose being directed into the PPP, which is involved in biomass production and maintenance of the cellular redox balance (Patra and Hay, 2014; Yamamoto et al., 2014). Since the pool size of PPP metabolites is small compared with those of glycolysis and the TCA cycle, we utilized ion chromatography connected to a Fourier transform orbitrap mass analyzer (IC-MS) (Hu et al., 2015). The amount of PPP metabolites, including 6-phosphogluconate (6-PG), ribose 5-phosphate (R-5-P) and sedoheptulose 7-phosphate (S-7-P), increased significantly from E8.5 to E9.5 (Fig. 6A). These results suggest that suppression of PFK-1/aldolase may increase the intracellular pool of PPP metabolites following CB.

We also found that the abundance of glycerol 3-phosphate (G-3-P) and glycine increased significantly from E8.5 to E10.5 (Fig. 6B,C). G-3-P and glycine are synthesized from the glycolytic metabolites dihydroxyacetone phosphate (DHAP) and 3-phosphoglycerate (3-PG), respectively. G-3-P is the starting material for glycerolipids, including the mitochondria-specific phospholipid cardiolipin (Houtkooper and Vaz, 2008). Therefore, the reduced amount of glycolytic intermediates (DHAP and 3-PG; Fig. 1) might be a result of redirection from the glycolytic pathway to the biosynthetic pathway, producing G-3-P and glycine.

### Oxygen conditions play a key role in glucose-derived carbon entry into the TCA cycle

**Fig. 7.**
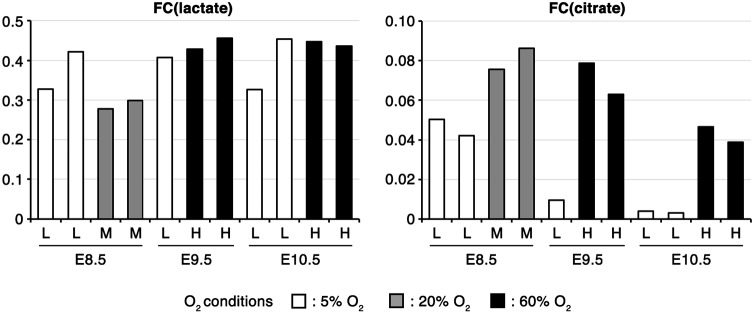
**Oxygen concentration** **affects glucose-derived carbon entry into the TCA cycle.** Fractional contribution of ^13^C from ^13^C_6_-glucose to lactate [FC(lactate)] or citrate [FC(citrate)] in *ex vivo* culture. After pre-culturing for 60-90 min under 5% (L), 20% (M) or 60% (H) O_2_ conditions, embryos were cultured with ^13^C_6_-glucose for 60 min. Each bar represents an independent sample. E8.5, ss 8-12; E9.5, ss 24-28; E10.5, ss 35-39.

CB is thought to promote maternal oxygen supply to embryos. To examine whether oxygen conditions affect the metabolic rewiring observed above, we performed labeling experiments with ^13^C_6_-glucose in whole-embryo culture (WEC). In WEC, E8.5 embryos require low oxygen conditions (5% O_2_ is optimal) for normal development, whereas at E9.5 or later the embryos require high oxygen conditions (60% O_2_ or higher is optimal) (Cockroft, 1990). Accordingly, we cultivated embryos under optimal oxygen conditions as well as under higher (20% for E8.5) or lower (5% for E9.5 and E10.5) oxygen conditions. FC(lactate) and the lactate pool size remained relatively constant between different oxygen conditions at all of the stages of development examined (Fig. 7, Fig. S4A,C). This suggests that glucose carbon flow into lactate is independent of oxygen concentrations. However, FC(citrate) and the citrate pool size were increased under higher oxygen conditions (E8.5, 20% O_2_; E9.5/E10.5, 60% O_2_; Fig. 7; Fig. S4B,D). These results suggest that CB stage embryos have the capacity to promote the entry of glucose-derived carbon into the TCA cycle depending on the oxygen supply.

### Lin28a as a possible intrinsic timing determinant of glucose metabolism rewiring

**Fig. 8.**
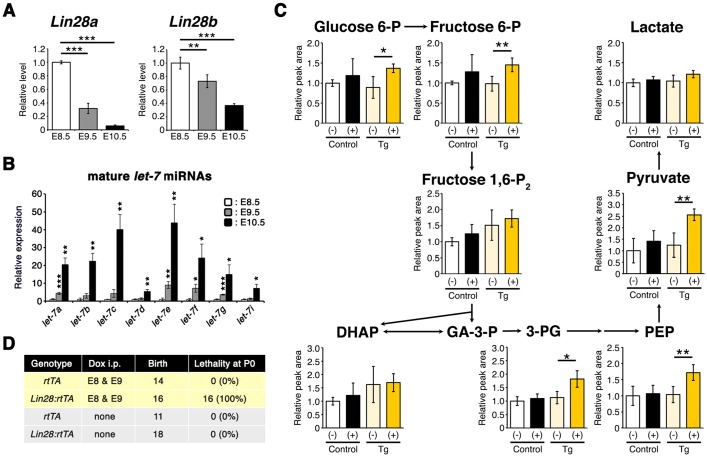
**Lin28a as a possible determinant of glucose metabolism rewiring timing.** (A) qPCR analysis of *Lin28a/b* mRNA. *Actb* was used as an internal control. *n*=4 independent sample replicates. **P<0.01, ***P<0.001 versus E8.5 (two-tailed Welch's *t*-test). E8.5, ss 10; E9.5, ss 23-26; E10.5, ss 34-36. (B) qPCR analysis of *let-7* miRNAs. U6 snRNA was used as an internal control. *n*=4 independent sample replicates. *P<0.05, **P<0.01, ***P<0.001 versus E8.5 (two-tailed Welch's *t*-test). E8.5, ss 9-11; E9.5, ss 24-26; E10.5, ss 34-36. (C) Measurement of glycolytic metabolites within *tetO-Lin28a:rtTA* E10.5 embryos by IC-MS. Dox was administered intraperitoneally at E8 and E9. Size-matched E10.5 embryos (ss 33-35) were used for analysis, and the relative peak area per embryo is shown. Control, *rtTA*; Tg, *tetO-Lin28a:rtTA*; (−), Dox non-treated; (+), Dox treated. *n*=4 independent sample replicates. *P<0.05, **P<0.01 (two-tailed Welch's *t*-test). (D) Lethality of *tetO-Lin28a:rtTA* P0 neonates treated with Dox at E8 and E9. Data are represented as mean±s.d.

The gradual decrease of *Aldoa* expression along the body axis raises the possibility that embryonic glucose metabolism rewiring is intrinsically regulated by the developmental program. We therefore focused on the heterochronic gene *Lin28a*, which regulates various cellular processes including embryonic stem cell self-renewal and glucose metabolism (Shyh-Chang and Daley, 2013; Shyh-Chang et al., 2013; Zhang et al., 2016). *Lin28a* mRNA is reported to be expressed throughout the embryo at E8.5, but gradually becomes restricted to the posterior part of embryos as development proceeds (Balzer et al., 2010), resembling the dynamic expression pattern of *Aldoa* during CB (Fig. S3). We confirmed that *Lin28a* mRNA expression dramatically decreases across the whole embryo from E8.5 to E10.5, whereas the expression of mature *let-7* microRNAs (miRNAs), which are negatively regulated by Lin28a, dramatically increased (Fig. 8A,B).

To examine whether the developmentally regulated expression of *Lin28a* is involved in the rewiring of embryonic glucose metabolism during CB, we used a conditional transgenic mouse line (*tetO-Lin28a:rtTA*) that overexpresses *Lin28a* in a doxycycline (Dox)-dependent manner (Fig. S5) (Zhu et al., 2010). *Lin28a* downregulation was prevented by overexpressing *Lin28a* via Dox treatment at E8.5 and E9.5. This resulted in a significant increase in some glycolytic intermediates downstream of the PFK-1 reaction (3-PG, phosphoenolpyruvate and pyruvate) in E10.5 embryos compared with non-treated embryos (Fig. 8C). The amount of PPP metabolites did not differ between *tetO-Lin28a:rtTA* E10.5 embryos with or without Dox treatment (Fig. S6). These observations suggest that the rewiring of embryonic glucose metabolism during CB is partly regulated by Lin28a. Furthermore, we found that perturbation of glucose metabolism rewiring by Lin28a overexpression during CB led to perinatal lethality at postnatal day (P)0 without obvious morphological defects (Fig. 8D).

Taken together, these results suggest that Lin28a is involved in the regulation of the timing of glucose metabolism rewiring following CB, which is essential for postnatal survival.

## DISCUSSION

**Fig. 9.**
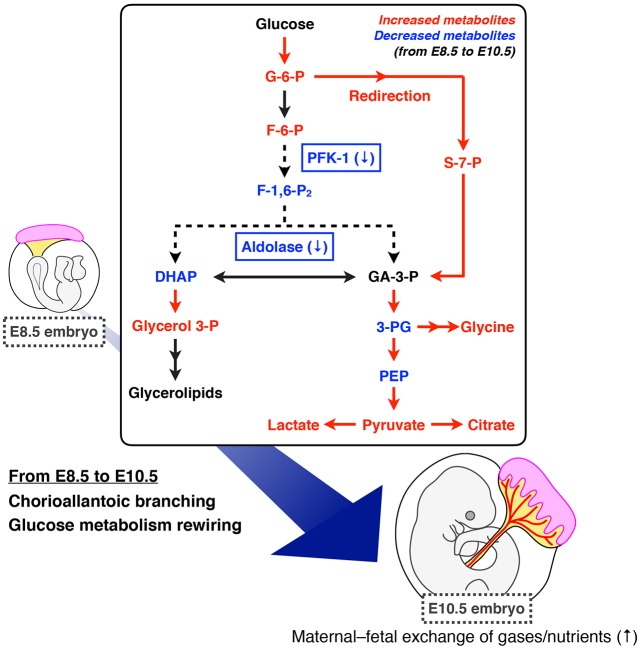
**Model of glucose** **metabolism rewiring during CB.** Our study suggests that embryos rewire glucose metabolism via suppression of PFK-1/aldolase to redirect glucose carbon flow into the PPP, which is partially controlled by downregulation of the heterochronic gene *Lin28a*.

Our global analysis of intracellular metabolites using metabolomics with mass-labeled glucose revealed that embryos have increased pools of lactate and citrate during CB, accompanying the increased fractional contribution of glycolysis to lactate biosynthesis. Furthermore, our data suggest that glucose carbon flow is redirected into the PPP via suppression of PFK-1 and aldolase during this stage of development (Fig. 9). These notions advance the classical view that embryos simply suppress glycolysis while accelerating the TCA cycle following CB. The observed increase in FC(lactate) can be explained by: (1) increased glycolysis, which is expected to result in increased lactate secretion; or (2) decreased lactate production from carbon sources other than glucose. Previous studies proposing decreased glycolysis relied on evidence that cultured embryos had reduced lactate secretion as development proceeded, a result that we reproduced (Fig. S7). However, measurement of lactate secretion alone is not indicative of glycolytic activity within embryos. The amount of secreted lactate can be strongly influenced by the surface area to volume ratio of the embryo, which decreases as the embryo enlarges substantially during CB. Additionally, the rate of lactate secretion and/or uptake may differ between distinct cell types. Consistent with this idea, our IMS analysis revealed an uneven distribution of lactate within embryos. Thus, future studies will address how glycolytic activity is coordinated with developmental progression at the tissue or cellular level.

Interestingly, the reduction of glycolytic intermediates precedes the changes in PFK-1/aldolase expression at E9.5. The enzymatic activity of aldolase first decreases from E8.5 to E9.5, but that of PFK-1 is suppressed only from E9.5 to E10.5. Suppression of both enzymatic activities at E10.5 could be attributed to the decreased expression of their genes, but the mechanisms of glycolytic flow suppression at E9.5 remain to be elucidated. PFK-1 activity is regulated by many allosteric inhibitors and activators, and thus allosteric regulations of PFK-1 could be involved in the glucose metabolism rewiring at E9.5. Citrate levels, a potent allosteric inhibitor of PFK-1, increased ∼2-fold from E8.5 to E9.5, suggesting an involvement in glucose metabolism rewiring. It is interesting to note that the expression of Pfkm mRNA, which is more sensitive to allosteric inhibition by citrate than Pfkl (Staal et al., 1987), increased at this stage (Fig. 5B). Although the enzymatic activity assay using cell lysates reflects not only the amount of enzymes but also, to some extent, the effects of endogenous metabolites (Yamamoto et al., 2014), such allosteric regulation of PFK-1 is presumably difficult to detect in the presence of excessive amounts of ATP, which is a substrate as well as an allosteric inhibitor of PFK-1. In addition, it should be noted that the enzymatic assay disrupts cellular structure and therefore does not detect the regulation of enzymatic activity according to cellular localization. This is important because some glycolytic enzymes, including Aldoa, are localized to specific cellular compartments in addition to the cytoplasm (Hu et al., 2016; Mamczur et al., 2013), and therefore regulation of the cellular localization of glycolytic enzymes could be one of the mechanisms that regulate glucose metabolism rewiring during CB.

Accompanying the onset of mammalian gastrulation and neurulation, the intrauterine environment of embryos changes following vitelline circulation development and CB, which enhances the maternal-fetal interaction for the exchange of gases and nutrients (Arora and Papaioannou, 2012; McGrath et al., 2003; Zohn and Sarkar, 2010). Given the close association between the timing of metabolic changes and CB, previous studies have suggested that the metabolic change was the result of a presumptive elevated oxygen supply to the embryos (Clough, 1985; Clough and Whittingham, 1983). Our ^13^C labeling experiment using WEC also showed that glucose-derived carbon entry into the TCA cycle is enhanced by high oxygen concentrations (Fig. 7), suggesting that embryos at the CB stage have the capacity to modulate the entry of glucose-derived carbon into the TCA cycle depending on the concentration of oxygen. However, our results also demonstrate that embryos at this stage sustain glucose carbon flow into lactate irrespective of the concentration of oxygen (Fig. 7). This implies that intrinsic developmental programs may be regulating glucose carbon flow through the glycolytic pathway irrespective of the presumptive increases in oxygen supply during CB. It should be noted that WEC might not recapitulate the metabolic state *in vivo* owing to differences in the environmental conditions surrounding the embryo, as suggested by inconsistencies between FC(citrate) and FC(lactate) *in vivo* and *ex vivo*. Nevertheless, our study suggests that WEC can be used to investigate the responsiveness of embryos to environmental challenges.

It is interesting to note that downregulation of *Aldoa* mRNA proceeds along the body axis during CB, implying regulation of glucose metabolism rewiring by a developmental program that also proceeds in a rostral-to-caudal direction (Stern et al., 2006). This view of glycolysis regulation by intrinsic factors, but not intrauterine environmental factors, is consistent with the idea that rapid embryonic growth during CB determines metabolic changes (New, 1978). In this regard, it is interesting to note that *Lin28a* expression is dramatically suppressed along the anterior-posterior axis from E8.5 to E10.5, similar to changes in the expression of *Aldoa*. *lin-28* was first identified as a heterochronic gene regulating developmental transitions in *C. elegans*; developmental events associated with specific larval stages were found to be skipped or reiterated upon the introduction of loss- or gain-of-function mutations in *lin-28*, respectively (Tsialikas and Romer-Seibert, 2015). Mammals have two homologs of *lin-28*, namely *Lin28a* and *Lin28b*. Although it is known that Lin28a deficiency impairs embryonic growth and, when combined with Lin28b deficiency, causes embryonic lethality at the mid-gestation stage, it has not been elucidated how Lin28a contributes to development at this stage (Shinoda et al., 2013). Interestingly, *Lin28a* knockout E10.5 mouse embryos show aberrant glucose metabolism represented by decreased levels of glycolytic intermediates downstream of the PFK-1 reaction (Shinoda et al., 2013). These metabolite changes are complementary to those induced by sustained expression of *Lin28a* in E10.5 embryos, suggesting that downregulation of *Lin28a* from E8.5 to E10.5 is involved in the regulation of glucose metabolism rewiring. Because the expression of PFK-1 and aldolase mRNAs was not affected by exogenous Lin28a overexpression at E10.5 (data not shown), how Lin28a downregulation controls embryonic glucose metabolism remains an open question.

Perturbation of glucose metabolism rewiring by suppression of *Lin28a* downregulation at the CB stage resulted in perinatal lethality, suggesting that metabolic rewiring is crucial for normal development. How these metabolic changes affect development during and after the CB stage remain to be elucidated and will be explored in future studies. Suppression of PFK-1 can divert glucose carbon flow into PPP in cancer cells and endothelial cells (De Bock et al., 2013; Yamamoto et al., 2014). This is also the case during embryonic development from E8.5 to E10.5, as the suppression of PFK-1 is accompanied by an increase in PPP metabolites such as S-7-P. Since PPP is important for NADPH production and *de novo* nucleotide synthesis, such rewiring of glucose metabolism might enable embryos to enhance biomass production while generating energy mainly through OXPHOS, thereby meeting increasing metabolic demands for the dramatic expansion in body mass, cell number and cell types from E9.5 onwards. In addition, the rewiring of glucose metabolism from E8.5 to E10.5 results in an increase in G-3-P and glycine, which can be further converted to glycerolipids and nucleotides, respectively. In addition to biomass production, redirecting glucose carbon flow into the PPP might potentiate embryonic antioxidant capacity by producing NADPH to counteract the oxidative stress associated with enhanced OXPHOS capacity at these stages, and also to avoid excessive cell death (L'Honoré et al., 2014). Consistent with these ideas, embryos during CB have been shown to be especially susceptible to PPP inhibition (Chamberlain and Nelson, 1963; McCandless and Scott, 1981; Turbow and Chamberlain, 1968).

Metabolic changes are known to precede cellular fate changes during the reprogramming of differentiated cells into induced pluripotent stem cells (Folmes et al., 2011), raising the possibility that metabolic rewiring constitutes a crucial part of the reprogramming/cellular differentiation processes. In addition, several lines of evidence suggest that enzymes and metabolites that are involved in glycolysis also function as signaling molecules (Chang et al., 2013; Ho et al., 2015; Lee et al., 2015; Luo et al., 2011; Yang et al., 2011). For instance, lactate promotes angiogenesis by activating the Raf-ERK pathway under hypoxic conditions in cancer cells (Lee et al., 2015). The *Lin28* genes act as gatekeepers between stemness and differentiation via regulation of both early proliferative cell fates and later differentiating cell fates (Faas et al., 2013; Polesskaya et al., 2007; Tsialikas and Romer-Seibert, 2015; Vadla et al., 2012; Yu et al., 2007). Therefore, downregulation of *Lin28a* might constitute a part of the developmental program that coordinates cell fate transitions with energy metabolic rewiring as a means to fulfill the changing metabolic demands of embryos for cell proliferation, cell differentiation, and morphogenesis during organogenesis.

The glucose metabolism rewiring observed in our study reflects changes in the whole embryo over time. Whether reorganization simultaneously occurs throughout the embryo, or whether there is a specific population of cells or a cell lineage that exhibits drastic metabolic rearrangement currently remains unclear. However, as discussed above, hurdles remain in studying metabolism within heterogeneous multicellular tissues. In this study, we demonstrated the potential of MALDI-IMS for metabolic studies during development as revealed by the unexpected distribution of lactate-rich tissues (i.e. the posterior/dorsal neural tube, somites and head mesenchyme in E9.5 embryos). Further technical advances, such as quantitative IMS analysis and genetically encoded metabolic probes to monitor metabolic pathway activity at the single-cell level *in vivo*, will aid in understanding how cellular metabolism contributes to development.

## MATERIALS AND METHODS

### Mice

Pregnant Jcl:ICR mice were purchased from CLEA Japan. *Col1a1-tetO-Lin28a* (Zhu et al., 2010) and ROSA26-rtTA*M2 (Hochedlinger et al., 2005) transgenic mice were obtained from the Jackson Laboratory. 9-tert-butyl doxycycline (Echelon) dissolved in water was administered to pregnant mice intraperitoneally at 0.25 mg/mouse. All experiments were performed with approval from the Animal Experiment Ethics Committee of the University of Tokyo, and in accordance with the University of Tokyo guidelines for the care and use of laboratory animals.

### Metabolic profiling of developing embryos by CE-MS

Embryos were dissected from the uterus and removed from yolk sac, amnion and allantois in cold PBS, and washed twice with 5% mannitol solution. Dissected embryos were immediately frozen with liquid nitrogen and stored at −80°C until use. Metabolite extraction was performed from the embryo with methanol containing an internal standard solution (Human Metabolome Technologies) as described (Soga et al., 2003). Metabolite quantities were determined by CE-MS (Agilent Technologies) from 11 (E8.5), 5 (E9.5) or 3 (E10.5) embryos/sample; *n*=3 independent sample replicates (Soga et al., 2006). PLS-DA was performed based on an algorithm described previously (Barker and Rayens, 2003). Multiple testing correction via false discovery rate (FDR) estimation was performed using R software (Storey, 2002).

### Tracing ^13^C_6_-glucose metabolic pathways in developing embryos by CE-MS

For whole-embryo culture (WEC) experiments, embryos were carefully isolated from the uterus with the yolk sac intact in prewarmed (37°C) dissection medium. Embryos were precultured for 60-90 min in DMEM containing 50% immediately centrifuged (IC) rat serum in a culture bottle placed in a rotational drum culture system (Ikemoto Rika, Tokyo, Japan). Then, ^13^C_6_-glucose (2 mg/ml final concentration) was added to the culture medium and the embryos were cultured for another 60 min. For intravenous injection, ^13^C_6_-glucose (24 mg/mouse) was administered to pregnant mice, and embryos were retrieved 60 min later. The amount of intracellular ^13^C-labeled metabolites in the embryos was determined by CE-MS as described previously (Sugiura et al., 2014). For details, see the supplementary Materials and Methods.

FC(citrate) and FC(lactate) were determined from the ^13^C labeling patterns of citrate and lactate acquired by CE-MS. The effects of naturally occurring isotopes were removed from the raw mass spectrometry data to obtain the corrected mass distribution vectors (MDVs) of the carbons in the metabolites (Kajihata et al., 2014; van Winden et al., 2002). Fractional contributions were calculated as described (Buescher et al., 2015). Although the small sample number prevents statistical testing, our data show a clear tendency for metabolic changes and embryonic response to environmental changes at the CB stage.

### MALDI-IMS

Embryos were dissected from the uterus with the Reichert's membrane and yolk sac intact in cold PBS. Dissected embryos were washed with 5% mannitol solution, embedded in Super Cryoembedding Medium (SCEM)-L1 (SECTION LAB, Hiroshima, Japan) and stored at −80°C until use. Frozen SCEM blocks in which embryos were embedded were sectioned at −16°C using a cryostat (Leica CM 3050) to a thickness of 8 µm. Kawamoto cryofilm was used to support fragile embryo tissues during cutting (Kawamoto, 2003). Sections were attached onto indium-tin oxide (ITO)-coated glass slides (Bruker Daltonics, Billerica, MA, USA) with electrically conducting double-adhesive tape (Shimadzu Corporation, Kyoto, Japan). Prepared tissues were coated with 9-aminoacridine as the matrix (10 mg/ml dissolved in 80% ethanol) (Benabdellah et al., 2009) by manually spraying with an artistic brush (Procon Boy FWA Platinum, Mr. Hobby, Tokyo, Japan). The matrix was simultaneously applied to multiple tissue sections in order to maintain consistent analyte extraction and co-crystallization conditions (Setou, 2010; Sugiura et al., 2009). MALDI imaging was performed using an Ultraflextreme MALDI-TOF mass spectrometer (Bruker Daltonics) and 7T FT-ICR-MS (Solarix Bruker Daltonics, Bremen, Germany) equipped with an Nd:YAG laser. Data were acquired in the negative reflectron mode with raster scanning by a pitch distance of 30 μm. Each spectrum was the result of 300 laser shots at each data point. In this analysis, signals between m/z 50 and 1000 were collected. In FT-ICR-MS (Fourier transform ion cyclotron resonance mass spectrometry) imaging, signals between m/z 100 and 400 were collected using the ʻcontinuous accumulation of selected ions' mode. Image reconstruction was performed using FlexImaging 4.1 software (Bruker Daltonics). The high mass accuracy provided by FT-ICR-MS allowed the selective ion signals of the metabolites to be obtained within a mass window of 5 ppm, enabling identification of the specific elemental composition of the compounds by comparing the highly accurate masses in databases (Marshall et al., 1998).

### Measurement of PPP metabolites by IC-MS

Trace amounts of PPP metabolites in developing embryos (*n*=4 independent sample replicates) were measured using an orbitrap-type mass spectrometer (Q-Exactive Focus, Thermo Fisher Scientific) connected to a high-performance ion chromatography (HPIC) system (ICS-5000+, Thermo Fisher Scientific) that enables highly selective and sensitive metabolite quantification owing to the ion chromatography separation and Fourier transform mass spectrometry principle (Hu et al., 2015). The sample preparation method was the same as for CE-MS analysis. For details, see the supplementary Materials and Methods.

### Lactate assay

Embryos were retrieved from the uterus in prewarmed (37°C) dissection medium. Embryos without the yolk sac, amnion and allantois were cultured under ambient oxygen conditions in DMEM containing 100 U ml^-1^ penicillin and 100 μg ml^-1^ streptomycin at 37°C in a 5% CO_2_ incubator (E8.5 and E9.5 embryos, 2 h; E10.5 embryos, 2.5 h; *n*=3 independent sample replicates). The amount of lactate released into the culture medium was determined using the Lactate Assay Kit (Biovision) following the manufacturer's protocol.

### Enzymatic activity assay

Activities of PFK-1 and aldolase were measured using a published protocol with modifications (Tian et al., 1998). Embryos [18-22 (E8.5), 4 (E9.5) or 1 (E10.5) embryo/sample; *n*=7 independent sample replicates] were sonicated in 0.1 ml lysis buffer containing 50 mM Tris acetate (pH 8.0), 10 mM dithiothreitol, 1 mM EDTA, 1 mM EGTA and Protease Inhibitor Mix (Roche), and then centrifuged at 20,000 ***g*** for 30 min at 4°C. The supernatant was used in the assays. The activities of PFK-1 and aldolase were determined by coupled enzyme assays, in which fructose 6-phosphate (a substrate of PFK-1) or fructose 1,6-bisphosphate (a substrate of aldolase) is added to the embryo lysates, and the resulting dihydroxyacetone phosphate from the substrate is evaluated by the rate of conversion of NADH to NAD^+^ by glycerol-3-phosphate dehydrogenase (GPDH), which is proportional to the activity of PFK-1 or aldolase; the conversion of NADH to NAD^+^ was measured by the decrease in absorbance at 340 nm by NanoDrop (Thermo Fisher Scientific). Samples were added to a cuvette containing buffer (50 mM Tris acetate, 2 mM Mg^2+^, pH 8.0) and enzymes [PFK-1 activity assay: aldolase, GPDH and triose phosphate isomerase (TPI); aldolase activity assay: GPDH and TPI; all purchased from Sigma]. Substrate concentrations for the PFK-1 activity assay: 1 mM ATP, 1 mM D-fructose 6-phosphate, 0.15 mM NADH; substrate concentrations for the aldolase activity assay: 2.5 mM D-fructose 1,6-bisphosphate, 0.15 mM NADH (all these substrates were from Sigma).

### Microarray and qPCR analysis

Total RNA was extracted from embryos using the RNeasy Plus Micro Kit (Qiagen) and RNA quality was checked using a Bioanalyzer (Agilent). Total RNA with RNA integrity number (RIN) values ≥7 was utilized for microarray analysis. One hundred nanograms of total RNA was used for analysis with the mouse GE 4×44K V2 microarray (Agilent) according to manufacturer's instructions. Data were analyzed using GeneSpring software (Agilent).

For qPCR, cDNA was synthesized from 200 ng total RNA using a PrimeScript RT Reagent Kit with gDNA Eraser (Takara). qPCR was performed with Takara SYBR Premix Ex Taq II (Tli RNaseH Plus) using a LightCycler 480 (Roche). Primers for qPCR are listed in Table S2. All primers gave a similar PCR amplification efficiency (∼2.0), allowing comparison of estimated gene expression levels among different genes using crossing point (Cp) values. For quantification of mature *let-7* microRNAs, cDNAs were synthesized using the TaqMan MicroRNA RT Kit (Applied Biosystems) from total RNA. RNA was extracted from embryos using Trizol (Invitrogen) and the mirVana miRNA Isolation Kit (Ambion). qPCR was performed with TaqMan Fast Advanced Master Mix (Applied Biosystems) using the LightCycler 480 system. We reproduced the result twice, and a representative result is shown in Figs 5 and 8.

### Mitochondrial DNA quantification

Embryos were lysed with lysis buffer containing proteinase K, and mitochondrial (mtDNA) and nuclear (nDNA) DNAs were purified by phenol-chloroform extraction [*n*=7 (E8.5) or *n*=4 (E9.5) independent sample replicates]. The amount of mtDNA and nDNA was quantified by qPCR using the following primers (5′-3′, forward and reverse): mtDNA, CCTATCACCCTTGCCATCAT and GAGGCTGTTGCTTGTGTGAC; nDNA (*Pecam1*), ATGGAAAGCCTGCCATCATG and TCCTTGTTGTTCAGCATCAC. We reproduced the result at least three times and a representative result is shown in Fig. 2.

### Western blotting

Western blotting to detect Lin28a and β-tubulin in E10.5 embryo extracts was performed as outlined in the supplementary Materials and Methods.

### Whole-mount *in situ* hybridization (WISH)

Detection of *Aldoa* by WISH at E8.5, E9.5 and E10.5 was performed as described in the supplementary Materials and Methods.
